# Rational Approach to New Chemical Entities with Antiproliferative Activity on Ab1 Tyrosine Kinase Encoded by the *BCR-ABL* Gene: An Hierarchical Biochemoinformatics Analysis

**DOI:** 10.3390/ph17111491

**Published:** 2024-11-06

**Authors:** Vitor H. da S. Sanches, Cleison C. Lobato, Luciane B. Silva, Igor V. F. dos Santos, Elcimar de S. Barros, Alexandre de A. Maciel, Elenilze F. B. Ferreira, Kauê S. da Costa, José M. Espejo-Román, Joaquín M. C. Rosa, Njogu M. Kimani, Cleydson B. R. Santos

**Affiliations:** 1 Biodiversity and Biotechnology Network of the Legal Amazon, Biotechnology Department, Federal University of Amapá, Macapá 68903-419, AP, Brazil; the.chemical.vh@gmail.com (V.H.d.S.S.); cleyson.cl@gmail.com (C.C.L.); luciaanebarros@hotmail.com (L.B.S.); igorsantosvictor@gmail.com (I.V.F.d.S.); 2 Graduate Program in Medicinal Chemistry and Molecular Modeling, Federal University of Pará, Belém 66075-110, PA, Brazil; 3 Laboratory of Modeling and Computational Chemistry, Department of Biological and Health Sciences, Federal University of Amapá, Macapá 68903-419, AP, Brazil; barrositb2008@hotmail.com (E.d.S.B.); alexandremaciell@yahoo.com.br (A.d.A.M.); elenilze.batista@ueap.edu.br (E.F.B.F.); 4 Graduate Program of Pharmaceutical Innovation, Federal University of Amapá, Macapá 68902-280, AP, Brazil; 5 Laboratory of Organic Chemistry and Biochemistry, University of the State of Amapá, Macapá 68900-070, AP, Brazil; 6 Computational Simulation Laboratory, Institute of Biodiversity, Federal University of Western Pará, Vera Paz Street, w/n Salé, Santarém 68040-255, PA, Brazil; kaue.costa@ufopa.edu.br; 7 Department of Pharmaceutical and Organic Chemistry, Faculty of Pharmacy, Campus of Cartuja, University of Granada, 18071 Granada, Spain; josemanuelespejo@correo.ugr.es (J.M.E.-R.); jmcampos@ugr.es (J.M.C.R.); 8 Natural Product Chemistry and Computational Drug Discovery Laboratory, Embu P.O. Box 6-60100, Kenya; njogu.mark@embuni.ac.ke

**Keywords:** chronic myeloid leukemia, drug design, imatinib, binding affinity, molecular docking

## Abstract

**Background:** This study began with a search in three databases, totaling six libraries (ChemBridge-DIVERSet, ChemBridge-DIVERSet-EXP, Zinc_Drug Database, Zinc_Natural_Stock, Zinc_FDA_BindingDB, Maybridge) with approximately 2.5 million compounds with the aim of selecting potential inhibitors with antiproliferative activity on the chimeric tyrosine kinase encoded by the *BCR-ABL* gene. **Methods:** Through hierarchical biochemoinformatics, ADME/Tox analyses, biological activity prediction, molecular docking simulations, synthetic accessibility and theoretical synthetic routes of promising compounds and their lipophilicity and water solubility were realized. **Results:** Predictions of toxicological and pharmacokinetic properties (ADME/Tox) using the top100/base (600 structures), in comparison with the commercial drug imatinib, showed that only nine exhibited the desired properties. In the prediction of biological activity, the results of the nine selected structures ranged from 13.7% < Pa < 65.8%, showing them to be potential protein kinase inhibitors. In the molecular docking simulations, the promising molecules LMQC01 and LMQC04 showed significant values in molecular targeting (PDB 1IEP—resolution 2.10 Å). LMQC04 presented better binding affinity (∆G = −12.2 kcal mol^−1^ with a variation of ±3.6 kcal mol^−1^) in relation to LMQC01. The LMQC01 and LMQC04 molecules were advanced for molecular dynamics (MD) simulation followed by Molecular Mechanics with generalized Born and Surface Area solvation (MM-GBSA); the comparable, low and stable RMSD and ΔE values for the protein and ligand in each complex suggest that the selected compounds form a stable complex with the Abl kinase domain. This stability is a positive indicator that LMQC01 and LMQC04 can potentially inhibit enzyme function. Synthetic accessibility (SA) analysis performed on the AMBIT and SwissADME webservers showed that LMQC01 and LMQC04 can be considered easy to synthesize. Our in silico results show that these molecules could be potent protein kinase inhibitors with potential antiproliferative activity on tyrosine kinase encoded by the BCR-ABL gene. **Conclusions:** In conclusion, the results suggest that these ligands, particularly LMQC04, may bind strongly to the studied target and may have appropriate ADME/Tox properties in experimental studies. Considering future in vitro or in vivo assays, we elaborated the theoretical synthetic routes of the promising compounds identified in the present study. Based on our in silico findings, the selected ligands show promise for future studies in developing chronic myeloid leukemia treatments.

## 1. Introduction

The development of new chemical entities (NCEs) with antiproliferative activity targeting the *BCR-ABL* gene is pivotal in advancing the treatment of chronic myeloid leukemia (CML). The *BCR-ABL* fusion gene, a result of the Philadelphia chromosome translocation between chromosomes 9 and 22, produces a constitutively active tyrosine kinase that drives the uncontrolled proliferation of leukemic cells [1]. Despite the clinical success of first-generation tyrosine kinase inhibitors (TKIs) such as imatinib, the emergence of resistance mutations and disease relapse remains a significant challenge, underscoring the need for novel therapeutic strategies [2,3,4].

In recent years, biochemoinformatics has emerged as a powerful approach in the rational design and discovery of NCEs. This interdisciplinary field combines computational chemistry, bioinformatics, and hierarchical data analysis to efficiently screen large chemical libraries, predict molecular interactions, and optimize lead compounds for enhanced specificity and efficacy [5,6]. By leveraging these advanced computational techniques, researchers can accelerate the drug discovery process and improve the precision of targeting oncogenic pathways, including *BCR-ABL*.

Recent studies underscore the potential of hierarchical biochemoinformatics in identifying and optimizing inhibitors against Abl tyrosine kinase encoded by the *BCR-ABL* gene. For example, structure-based virtual screening coupled with molecular dynamics simulations has been utilized to discover potent inhibitors capable of overcoming resistance mutations [7,8]. Additionally, machine learning algorithms have been increasingly applied to predict the antiproliferative activity of compounds, thus facilitating the selection of the most promising candidates for experimental validation [9,10,11]. These approaches not only streamline the identification of effective NCEs but also provide insights into their mechanisms of action at the molecular level.

Moreover, the integration of hierarchical clustering techniques allows for the systematic categorization of chemical entities based on their structural and functional characteristics. This method enhances the ability to identify key molecular features that contribute to the antiproliferative activity against *BCR-ABL*, thereby guiding the rational design of more potent and selective inhibitors [12,13,14]. Such hierarchical approaches are instrumental in prioritizing compounds for further development and clinical testing.

In this study, we aim to employ an hierarchical biochemoinformatics analysis to discover and optimize new chemical entities with antiproliferative activity targeting the *BCR-ABL* gene. Our methodology involves a multi-step process: initial virtual screening to identify potential lead compounds, followed by molecular docking simulations to assess their binding affinities and stability.

**Figure 1:**
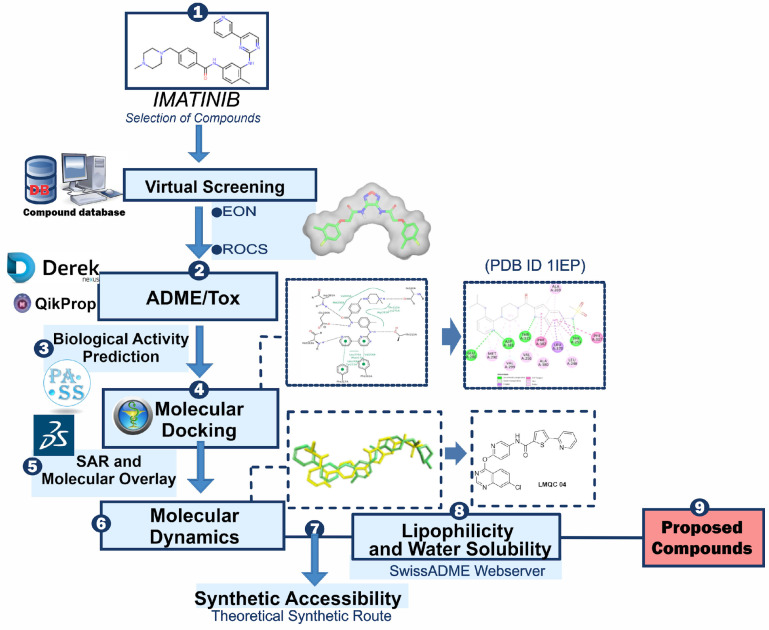
General scheme summarizing the methodological steps.

Subsequently, machine learning models will predict the biological activity of these compounds, and hierarchical clustering will be used to categorize and refine the candidates based on their structural and functional properties. Through this comprehensive approach, we seek to enhance the efficiency and effectiveness of the drug discovery process, ultimately contributing to the development of novel therapeutics for CML. By integrating state-of-the-art computational tools and data-driven methodologies, this study aims to address the current challenges in targeting *BCR-ABL* and to provide new insights into the design of effective antileukemic agents. The general scheme summarizing the methodological steps in this paper is shown in Figure 1.

## 2. Results and Discussion

Ligand-based virtual screening was performed using the reference compound imatinib, due to its crystallography being defined for tyrosine kinase antiproliferative activity (PDB ID: 1IEP) in three commercial compound databases (ChemBridge-DIVERSet, ChemBridge-DIVERSet-EXP, Zinc_Drug Database, Zinc_Natural_Stock, Zinc_FDA_BindingDB and Maybridge), using the ROCS (Rapid Overlay of Chemical Structures) program v. 3.6.2.0, by screening using shape similarity. This screening resulted in a total of 2000 structures per database, totaling 12,000 structures based on the pivotal compound. The structures obtained by ROCS screening were subjected to a new virtual similarity screening based on electrostatic affinity with the pivot molecule, imatinib, using the EON program, v. 3.0.0.0. This provided the “Top100” per database, resulting in 600 structures that advanced to pharmacokinetic and toxicological predictions.

### 2.1. Pharmacokinetic Properties Prediction

At this stage, the 600 structures obtained from the ligand-based virtual screening of the EON software were subjected to pharmacokinetic and subsequently toxicological activity prediction. Not all molecules will have biological antiproliferative activity for tyrosine kinase, due to unsatisfactory pharmacokinetic properties or presence of toxicophoric groups. At this stage, efficient filter selection is essential to access the best pharmacokinetic results for the development of future promising drugs.

Among the filters used to evaluate the pharmacokinetic profile are #stars or “drug-like”, molecular weight (MW), Solvent Accessible Surface Area (ASAS) along with its hydrophobic component (HFOAS) and hydrophilic component (HFIAS), molecular volume (MV), number of hydrogen bond acceptors (HBOs) and donors (DLHs), n-octanol/water partition coefficient (logP), solubility parameter (logS), predicted Caco-2 cell permeability, blood-brain barrier partition coefficient (logCS), skin permeability (logKp), number of predicted primary metabolites (#metab), percent human oral absorption (%OHA), and polar surface area (PSA) [15]. 

Using the parameters stipulated by Malolo et al. (2015) [15], a total of 178 structures were selected after pharmacokinetic analysis of results from the six commercial compound databases (ChemBridge-DIVERSet, ChemBridge-DIVERSet-EXP, Zinc_Drug Database, Zinc_Natural_Stock, Zinc_FDA_BindingDB and Maybridge). All candidates were compared with the values obtained for imatinib, the reference compound, in order to obtain the molecules with the best pharmacokinetic profiles which then passed to the toxicological prediction stage via Derek Nexus v. 4.1.0 software.

The software was used to find plausible, probable or certain alerts for carcinogenicity, mutagenicity, genotoxicity, hepatotoxicity and teratogenicity. The results of this prediction, which can be seen in Table 1, resulted in molecules that did not present any toxicity alerts. These were selected as promising drugs for the later stage of biological activity prediction, which was performed via the Pass Online Prediction server [16,17].

It is noteworthy that the pivotal compound, imatinib, presented a “plausible” alert for methemoglobinemia, as it contains compounds that will probably be metabolized or hydrolyzed to form a simple aniline, which is the cause of this activity in humans, rats, and mice. This fact reinforces the choice of the molecules resulting from the screening as candidates for the subsequent stages [18]. This alert may be the cause of the most reported adverse reactions (>30%): edema, nausea, vomiting, muscle cramps, musculoskeletal pain, diarrhea, rash, fatigue, and abdominal pain [19]. The pharmacokinetic properties analyzed were observed according to the range of values accepted for drugs described by Malolo et al. (2015) [15], as can be seen in Table 2, and limits were defined close to those indicated by the pivotal molecule, imatinib.

The computational study of ADME parameters is a fundamental tool for drug discovery, selection and development of bioactive compounds with a view to preclinical and clinical studies due to its speed, low cost and reduction in the number of experiments involving animals [20,21,22].

All compounds submitted to pharmacokinetic predictions presented #star = 0, which indicates that all calculated parameters were within the recommended range for 95% of known drugs. The overall ADME conformity index, drug similarity parameter (indicated by #stars), was used to evaluate the pharmacokinetic profiles of the isolated compounds. The #stars parameter indicates the number of property descriptors calculated by QikProp v. 2014-3 [23] that are outside the optimal range of values for 95% of known drugs.

The blood-brain barrier is a specific structure that protects, controls and regulates the homeostasis of the central nervous system by separating the brain from systemic blood. For a drug with biological activity in the CNS, a high penetration value is necessary. However, this study seeks structures without activity in the CNS; thus, they should present low penetration values, minimizing side effects [24]. The values for the predictions of the brain/blood partition coefficient (QPlogCS) should be less than 1, for molecules inactive in the CNS. Therefore, in this analysis, the structures that presented the lowest values were selected, which were the nine structures highlighted in Table 2.

The values for %OAH are above 85% for all structures, values considered high, according to the literature [15]. Properties such as HFOAS and HFIAS were within the acceptable range for all selected structures. When comparing the values obtained from the in silico study for certain pharmacokinetic properties to experimental results of imatinib deposited on the Drug Bank website [25], we can see that the software obtained approximate results of %OAH (98%), CNS (0.7624) and HFIAS (86.28). These approximate values guarantee credibility to the methodology used, reinforcing the use of the filters chosen to select possible drugs for the subsequent stage.

### 2.2. Biological Activity Prediction

After toxicological prediction, the four selected compounds (Table 3) were analyzed for potential biological activity using the PASS Online server [17]. The software assesses the overall biological potential of a drug-like organic molecule. PASS provides simultaneous predictions of many types of biological activity based on the structure of organic compounds. Thus, PASS can be used to estimate biological activity profiles for molecules before their chemical synthesis and biological testing. It provides the studied compound with a Pa value (probability of being active) that estimates the chance that the studied compound belongs to the subclass of active compounds (it resembles the structures of molecules, which are the most typical in a subset of “active” compounds in the PASS training set), and a Pi value (probability of being inactive) that estimates the chance that the studied compound belongs to the subclass of inactive compounds (it resembles the structures of molecules, which are the most typical in a subset of “inactive” compounds in the PASS training set) [26].

For a compound to show potential anticancer activity, it needs to eliminate cancer cells while leaving normal tissue unharmed. Recent studies have developed drug classes including: antimetastatic agents, which alter malignant cell surfaces to reduce metastatic potential; biological response modifiers, which adjust metabolic and immune responses; and antineoplastic agents, which inhibit tumor growth and destroy cancer cells [27].

The prediction of biological activity of the four compounds obtained in the screening presented biological activity values for inhibition of tyrosine kinase activity and BCR-ABL related to the emergence of proliferative activity of CML. The Pa and Pi values can be seen in Table 3. Compound LMQC04 had the most satisfactory result, coming close to the values of the pivot compound, with compounds LMQC01 and LMQC04 being selected for molecular docking study.

### 2.3. Molecular Docking Study

According to the literature, the binding mode prediction using docking should present an RMSD value lower than 2.0 Å in the crystallographic pose of the ligand [28,29,30,31,32]. Therefore, these results with the methodological proposal using these parameters are satisfactory. We emphasized in our previous study the use of molecular docking tools to search for new potential leads or hits [33,34,35,36,37].

**Figure 2:**
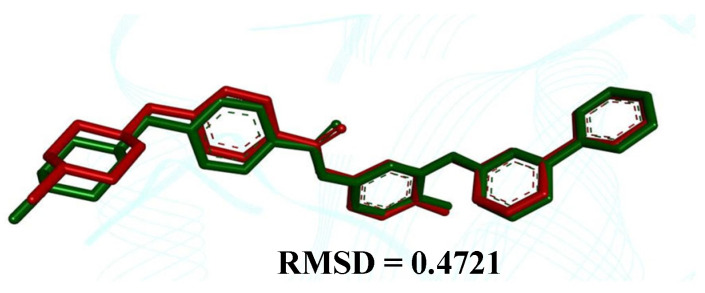
Superpositions of the ligand with crystallographic pose (in red) with the calculated poses (in green)—Abl Kinase Domain (organism *Mus musculus*, PDB ID 1IEP), showing an RMSD value equal to 0.4721 Å.

The comparison between the crystallographic ligand imatinib (red color) and the best conformation predicted by molecular docking (green color) can be seen in Figure 2, which shows the pose with an RMSD value of 0.4721 Å.

Redocking showed a binding affinity (ΔG) of −13.03 kcal mol^−1^, close to the experimental value of −11.18 kcal mol^−1^, with a variation of ±1.85 kcal mol^−1^. This indicates that our docking protocol is effective for evaluating the molecular binding mode of this type of complex (see Table 4).

**Figure 3:**
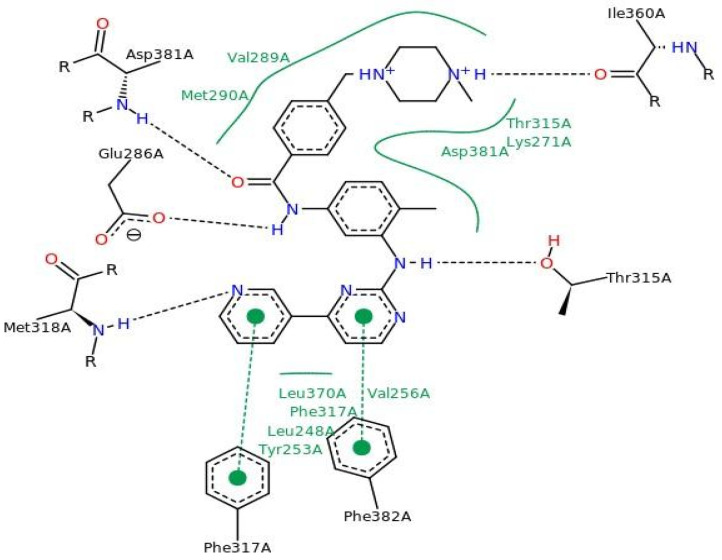
Interactions of imatinib with key amino acid residues in the active site of the Ab1 kinase domain.

Figure 3 shows the molecular docking results for imatinib (PDB ID 1IEP), with interaction sites around the alpha-helix (residues Leu348 to Glu286-Met290) and the beta-sheet (residues Tyr253-Val256, Thr315-Met318, Ile360, Leu370, and Ala380-Phe382). Hydrogen bonds were observed with residues Glu286, Thr315, Met318, Ile360, and Asp381, supporting data from literature studies [38].

**Figure 4:**
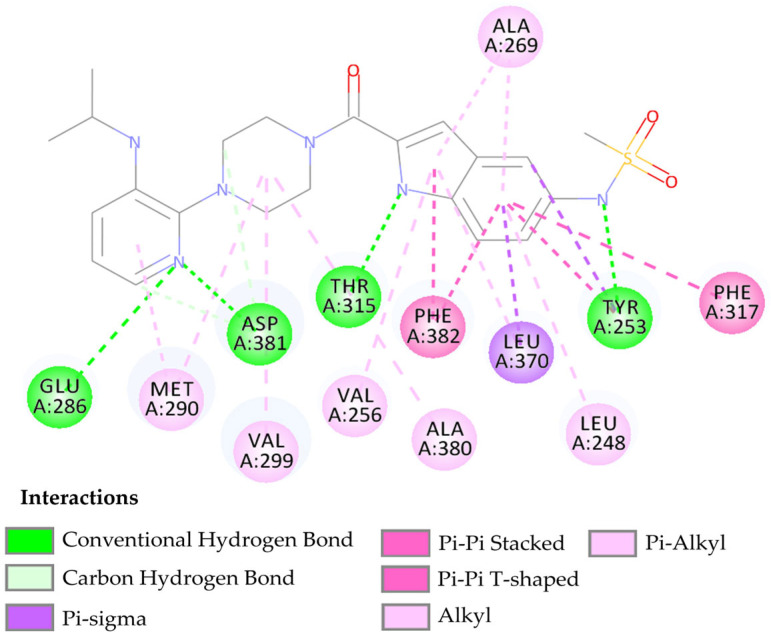
Predicted interactions between the BCR-ABL tyrosine kinase active site and compound LMQC01.

The compound LMQC01 presented a binding affinity value of −8.6 Kcal mol^−1^; in relation to the pivot compound, this result was below expectations. In the molecular docking study, it presented four hydrogen interactions with Tyr253, Thr315, Glu286 and Asp381, seven pi-alkyl interactions with the amino acids Leu248, Val256, Ala269, Ala280, Met290, and Val299, and finally, two pi-pi interactions paired with Phe382 and Phe317. For compound LMQC01, interactions around the alpha helix between amino acid residues Leu248 and Glu286-Met290 were observed in the active site of the enzyme. Interactions with amino acid residues Tyr253, Thr315 and Ala381-Phe382 are located around the beta sheet, when compared with the imatinib molecule in the active site of the protein (see Figure 4 and Figure S1 in Supplementary Materials).

**Figure 5:**
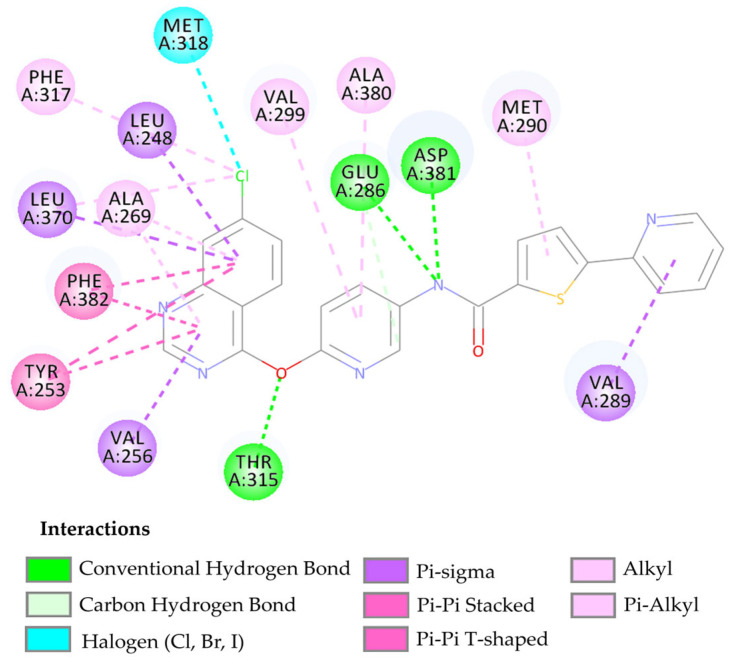
Predicted interactions between the active site of BCR-ABL tyrosine kinase and the compound LMQC04.

The compound LMQC04 presented a binding affinity value close to imatinib (−12.2 Kcal mol^−1^); in relation to the others, this was the most satisfactory result. It presented three hydrogen interactions with Thr315, Glu286 and Asp380, four pi-sigma interactions with the amino acids Leu248, Leu370, Val256 and Val289, five π-alkyl interactions with Phe317, Val299, Ala380, Met290, and Ala269, and finally, two pi-pi interactions paired with Phe382 and Tyr253, totaling 14 interactions (see Figure 5 and Figure S2 in the Supplementary Materials).

After performing the molecular docking study and comparing the results with the template compound, the interaction around the alpha-helix of the protein occurs with residues Leu248, Glu286-Met290, while in the beta sheet, it occurs between residues Tyr253-Val256, Thr315-Met318, Leu370, and Ala380-Phe382. These interactions demonstrate that the compound LMQC04 has its main interactions in the active site of the protein, leading to its consideration as a promising drug candidate (see Figure 5).

Quantitative data on the types of interactions and their respective distances and binding affinities between the ligands and the BCR-ABL tyrosine kinase receptor are shown in the Supplementary Materials. It was verified that, among the structures, the increase in the diversity of interactions with different amino acids can result in a decrease in the binding affinity values, which indicates a greater degree of spontaneity of the interactions.

Interactions with the residue Thr315 occurred in the LMQC01 and LMQC04 structures in addition to imatinib. Interactions with the amino acid Leu370 occurred only in the LMQC04 structure. Interactions with the amino acid Ala269 also occurred in two structures. Interactions with the amino acid Val269 occurred in one structure and with Tyr253 occurred in two structures. Interactions with the amino acids Glu286 and Met290 occurred in two structures. These amino acid residues presented the highest number of interactions, indicating that they may have important relevance for inhibitory activity. The structures presented three or more hydrogen bond interactions, which may be a cause of the decrease in the electron affinity values, since this type of interaction between the receptor and the ligand indicates the stabilization of the complex, being responsible for the stability of the bioactive conformations.

All compounds tested presented satisfactory binding affinity values, with emphasis on the compound LMQC04 (−12.2 kcal mol^−1^). This compound presented binding affinity close to imatinib; however, in the toxicological screening, there was no alert for toxicophoric groups. This compound was selected as a promising candidate for antiproliferative activity in CML cells with possibly fewer adverse effects. After analyzing the results, an additional study was performed to investigate Structure–Activity Relationship (SAR), Molecular Overlay, Synthetic Accessibility and Theoretical Synthetic Route of Promising Compounds and Lipophilicity and Water Solubility.

### 2.4. Molecular Dynamics Simulations

**Figure 6:**
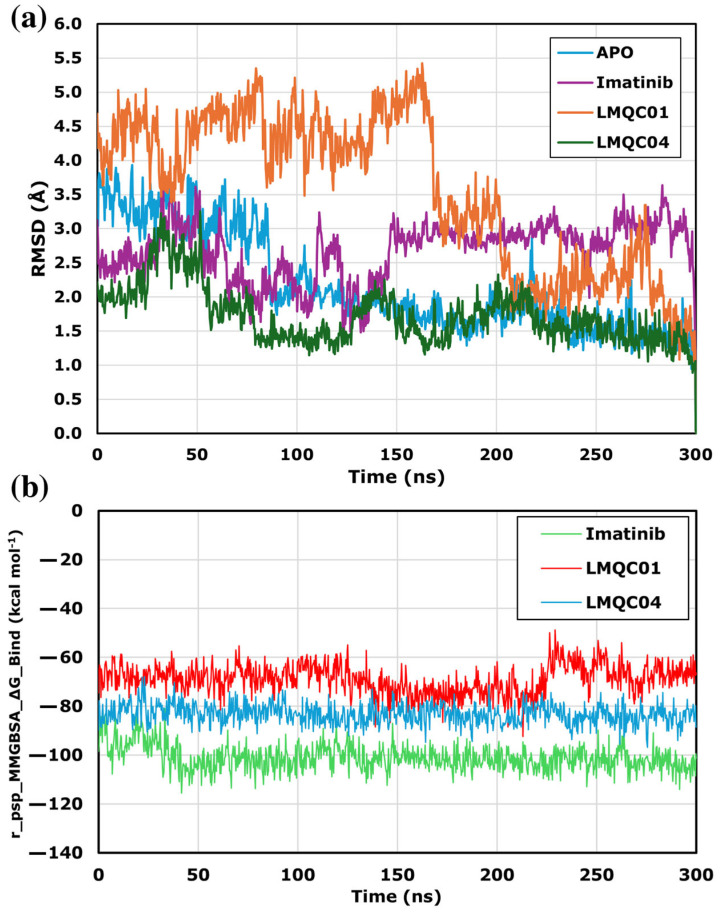
RMSD alignment analysis among apo-protein and ligand-complexes for C-Abl kinase domain (PDB ID: 1IEP) (**a**) based on 300 ns MD analysis. MMGBSA_∆G_Binding value line chart for 300 ns MD simulation (**b**).

In Molecular Dynamics (MD), the dynamic behavior of a molecular system is simulated to assess protein-ligand complex stability. MD simulations were performed using the Desmond module of Schrödinger, and the resulting trajectories were analyzed with the Simulation Interaction Diagram. Figure 6a shows the protein’s Root Mean Square Deviation (RMSD), indicating conformational changes in the complex compared to the apo-protein. The LMQC01 complex presented higher RMSD values in the first 168 ns in comparison to the other complexes studied, and presented lower RMSD values, remaining stable in the last 132 ns. Although the LMCQ01 complex deviates from the apo-protein, the maximum difference was about 3.6 Å. The imatinib and LMQC04 complexes remained stable, with RMSD changes under 2.2 Å throughout 300 ns. Since all protein–ligand complexes showed stable RMSD values, MM-GBSA binding energy was used for further analysis.

The Molecular Mechanics energies combined with the Generalized Born and Surface Area continuum solvation (MM-GBSA) line plots (Figure 6b) presented for ligands interacting with protein target C-Abl kinase provide insights into the binding affinity over time during molecular dynamics simulations. The relatively lower r_psp_MMGBSA_∆G_Binding value suggests stronger binding affinity, which is a desirable characteristic for potential inhibitors. All ligands demonstrate consistently low binding free energy throughout the 300 ns simulation, indicating a stable interaction with the target. The reference compound imatinib showed the lowest binding energy values, ranging from −115.62 to −80.72 kcal mol^−1^, with a mean of −101.03 kcal mol^−1^. For LMQC04, the binding energy was consistent around −96.80 to −67.98 kcal mol^−1^, with a mean of −83.25 kcal mol^−1^ which is more stable and has lower energy than LMQC01, which presented an energy variation between −92.36 to −48.77 kcal mol^−1^, with an average of −69.00 kcal mol^−1^.

**Figure 7:**
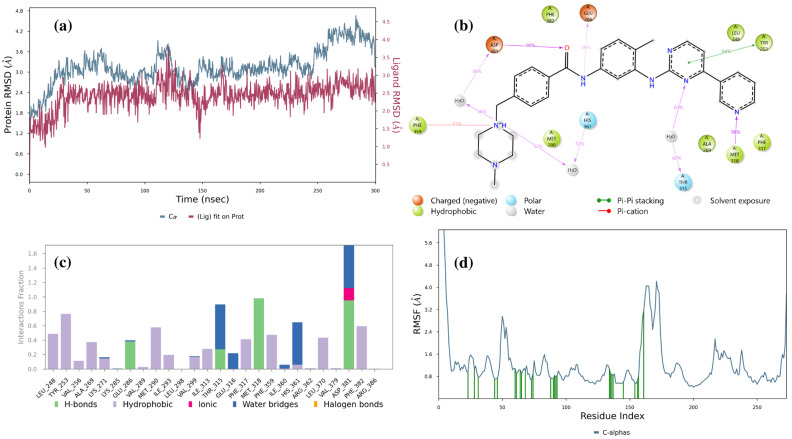
Result of 300 ns MD analysis for imatinib binding to the Abl-kinase domain. The protein–ligand RMSD plot of imatinib bound to the Abl-kinase domain (**a**) (PDB ID: 1IEP). Ligand–protein contact interactions scheme with the protein residues of imatinib bound to Abl-kinase (**b**). Protein–ligand contacts histogram of the interaction fraction of H-bond (green), hydrophobic bond (purple), ionic bond (magenta), and water bridges (blue) for imatinib (**c**). RMSF plot of imatinib (**d**) protein–ligand complex.

Detailed analyses regarding imatinib, LMQC01 and LMQC04 are shown below. Figure 7a represents the imatinib ligand in complex with the Abl-kinase domain; the RMSD values of the protein (blue line) fluctuate around a relatively narrow range (with a minimum value of 1.57 Å, and a maximum of 4.66 Å), suggesting that the protein maintains a stable conformation throughout the simulation. The ligand RMSD also shows limited fluctuation (with a minimum value of 0.97 Å, and a maximum of 3.83 Å), indicating that once bound, the ligand remains consistently positioned within the binding site of the enzyme.

Figure 7b shows that Asp381 forms an H-bond with amide oxygen 94% of the time. Asp381 also forms an H-bond with a piperazine nitrogen atom and water 56% of the time. Glu286 forms an H-bond with the nitrogen atom of the amide group 38% of the time. His361 forms an H-bond with the piperazine nitrogen atom and water 52% of the time. Thr315 forms an H-bond with a nitrogen atom of the pyrimidine ring 60% of the time. Met318 forms an H-bond with the nitrogen atom of pyridine 98% of the time. 

The histogram indicates that the dominant bonds are H-bond and water bridge; Glu286, Thr315, Met318, and Asp381 were major protein–ligand interaction involvers (Figure 7c). The Root Mean Square Fluctuation (RMSF) plot (Figure 7d) indicates protein behavior during the ligand binding process. The relatively low RMSF value suggest that the presence of the ligand does not lead to significant increases in structural flexibility, indicating stability.

**Figure 8:**
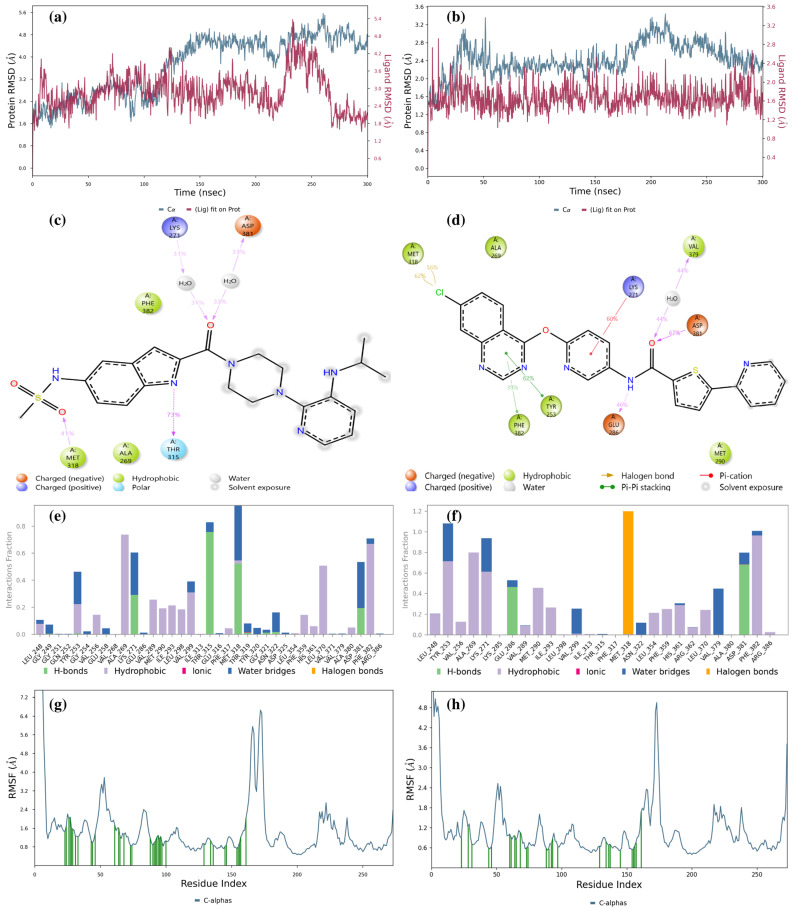
Result of 300 ns MD analysis for LMQC01 and LMQC04 binding to the Abl-kinase domain. The protein–ligand RMSD plot of LMQC01 (**a**) and LMQC04 (**b**) bound to the Abl-kinase domain (PDB ID: 1IEP). Ligand–protein contact interactions scheme with the protein residues of LMQC01 (**c**) and LMQC04 (**d**) bound to Abl-kinase. Protein–ligand contacts histogram of the interaction fraction of H-bond (green), hydrophobic bond (purple), ionic bond (magenta), water bridges (blue), and halogen bonds (orange) for LMQC01 (**e**) and LMQC04 (**f**). RMSF plot of LMQC01 (**g**) and LMQC04 (**h**) protein–ligand complex.

Figure 8a shows that the LMQC01 ligand complexed with the Abl kinase domain displays more RMSD fluctuation in both protein and ligand compared to the Abl kinase–imatinib complex, yet remains within a narrow range, indicating stable interactions with some structural flexibility. In the first 125 ns, protein and ligand RMSD values overlap. Figure 8b shows the LMQC04 ligand with the Abl kinase domain, where protein RMSD remains low and stable, suggesting minimal deviation from its initial conformation. The ligand’s stable RMSD suggests consistent interaction with the protein, with RMSD differences remaining within 1.4 Å, indicating strong stability in both complexes. These low and stable RMSD values suggest that LMQC01 and LMQC04 form stable complexes with the Abl kinase domain, potentially inhibiting enzyme function.

Figure 8c illustrates Ligand-Protein Contacts for LMQC01: Lys271 and Asp381 form H-bonds with an amide group and water 31% and 33% of the time, respectively. Met318 forms an H-bond with the sulfonamide group 41% of the time, and Thr315 forms an H-bond with the indole group 73% of the time. 

For LMQC04 ligand interactions (Figure 8d), it is observed that Glu286 forms an H-bond with the nitrogen atom of the amide group in the ligand structure 46% of the time. Asp381 forms an H-bond with the amide oxygen in the ligand structure 67% of the time, and Val379 forms an H-bond between amide oxygen and water 44% of the time. In Figure 8e,f, we can still observe the presence of the H-bond, water bridge, hydrophobic, and halogen bonds, which will tend to make the complexes stable.

RMSF plots show each protein residue’s flexibility during the simulation; lower values indicate rigidity, while higher values indicate flexibility. Our RMSF data (Figure 8g,h) reveal the protein’s dynamic behavior upon ligand binding. 

For both LMQC01 and LMQC04 complexes with the Abl kinase, RMSF values suggest that ligand presence does not increase structural flexibility, which would imply destabilization. Instead, the ligands appear to maintain or enhance enzyme stability. Additional MD simulation details are provided in Supplementary Figures S3–S7 and Table S4.

### 2.5. Structure–Activity Relationship (SAR) and Molecular Overlay

The LMQC01 and LMQC04 molecules were looked up in the PubChem database [41] to obtain data on their biological activities. The LMQC01 molecule is delavirdine, a non-nucleoside reverse transcriptase inhibitor with activity specific for HIV-1. The study identified that this molecule has already been patented as an antileukemic agent.

The different selection approaches are justified by their efficiency, potential for therapeutic innovation, and strategic alignment with current trends in pharmaceutical development. This approach accelerates the discovery of new treatments by maximizing the use of pre-existing drugs, and contributing significantly to the fight against leukemia. 

The LMQC04 molecule does not have any patents filed. It is only reported that it has antiviral activity (against dengue virus 2 and bovine viral diarrhea virus 1) and cytotoxic activity for the BHK-21 and A-549 cell lines [41]. The LMQC01 and LMQC04 molecules were subjected to molecular overlay with the pivot molecule, aiming to measure their steric and electrostatic similarity (see Table 5).

The overlay results showed that both molecules have a similarity of 41 to 50 est (50% steric contribution), see Table 5. For 70 est and 100 est (70% steric contribution and 100% steric contribution, respectively) there was a very small variation in the values obtained. The values ranged from 57% to 58% for 70 est and 82% to 83% for 100 est (see Table 5).

**Figure 9:**
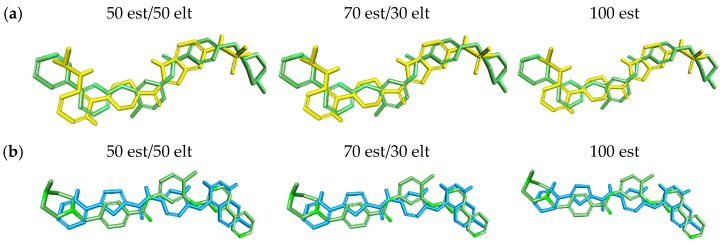
Graphical representation of the molecular overlay analysis between molecules (**a**) LMQC01 (yellow) and (**b**) LMQC04 (blue) with the reference molecule (imatinib—green).

The results obtained demonstrate the great similarity between the superimposed molecules and the pivot, as visualized in the graphical representation of the molecular overlay (see Figure 9).

### 2.6. Synthetic Accessibility

The synthetic accessibility of imatinib, LMQC01 and LMQC04 was predicted using SwissADME and AMBIT-SA to obtain a more accurate parameter (see Table 6). The results via SwissADME indicated that all compounds have Synthetic Accessibility Score (SA) compatible with easy difficulty syntheses. 

However, the SA score obtained by the LMQC and pivot molecules are compatible with synthesis of medium difficulty. Therefore, these results together indicate that synthetic accessibility is not an obstacle to the large-scale production of the compounds studied.

### 2.7. Theoretical Synthetic Routes Proposed to Compounds LMQC01 and LMQC04

Compound **LMQC 01** can be synthesized according to the procedure carried out by O. S. Pedersen et al. [42]. A coupling reaction between piperazine **I** and indole carboxylic acid derivative **II** using 1-ethyl-3-(3-(dimethylamino)propyl)carbodiimide (EDC) as activating agent will produce intermediate **III**. 

**Figure 10:**
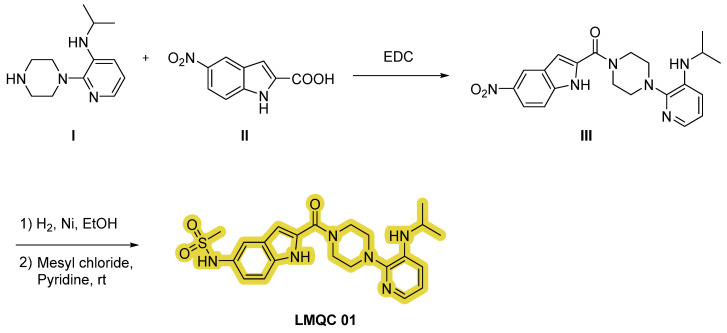
Synthetic route of the compound **LMQC 01**. Starting materials **I** and **II** are commercially available.

Finally, through a sulfonylation reaction with mesyl chloride and pyridine with previous reduction of the nitro moiety of derivate **III** using hydrogen (H_2_) and nickel (Ni) in ethanol (EtOH), the desired compound **LMQC 01** will be produced (Figure 10).

**Figure 11:**
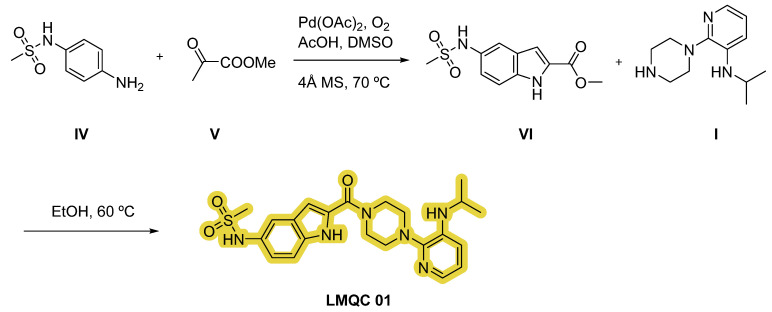
Alternative synthetic route of the compound **LMQC 01**. 4 Å MS (molecular sieves). Starting materials **I**, **IV** and **V** are commercially available.

Furthermore, we have designed an alternative strategy based on the construction of indole derivative **VI** with aniline **IV** and ketone **V** via aerobic cross-dehydrogenative coupling using palladium (II) acetate (Pd(OAc)_2_) as catalyst, oxygen as oxidant, acetic acid (AcOH) and dimethyl sulfoxide (DMSO) as solvents. Subsequently, reaction of intermediate **VI** and substituted piperazine **I** in EtOH will originate the target compound **LMQC 01** [43] (Figure 11).

**Figure 12:**
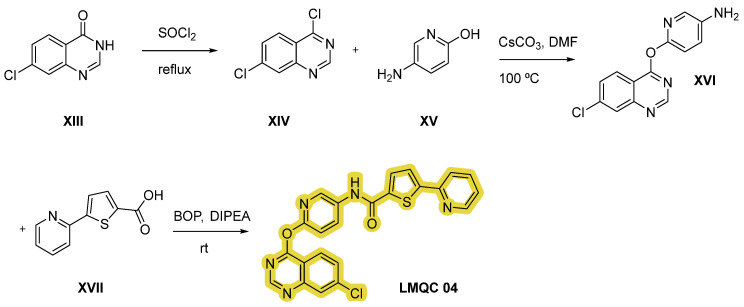
Synthetic route of the compound **LMQC 04**. Starting materials **XIII, XV** and **XVII** are commercially available.

Compound **LMQC 04** can be obtained by a method reported in previous work of E. S. Leal et al. [44] (Figure 12). Treatment of quinazoline derivative **XIII** with excess of thionyl chloride (SOCl_2_) under reflux conditions will originate double chlorinated quinazoline **XIV**. Secondly, nucleophilic reaction between intermediate **XIV** and aminopyridinol **XV** using cesium carbonate (CsCO_3_) as a base and DMF as solvent will give derivative **XVI**. Lastly, the target carboxamide **LMQC 04** will be obtained by amidation with carboxylic acid **XVII** moderated by DIPEA and 1-benzotriazolyl-oxytris-(dimethylamino)phosphonium-hexafluoro-phosphate (BOP) as coupling reagents.

### 2.8. Prediction of Lipophilicity and Aqueous Solubility via SwissADME Webserver

The predicted LogP values via SwissADME [45,46,47] for the two selected molecules and imatinib are shown in Table 7. LogP is considered an important ADME descriptor. It is a parameter used to express how lipophilic a given molecule is [45]. This property affects the tendency of a compound to decompose in non-polar environments versus aqueous environments. Increased lipophilicity can generally lead to increased permeability, protein binding and volume of distribution [48,49,50].

For a more assertive analysis, it is necessary to consider the consensus LogP, as it is an average of the predictions of the other five methods. Therefore, the values obtained for LMQC01 and LMQC04 were 1.75 and 4.29, respectively, while imatinib resulted in 3.38. It is worth mentioning that such positive values indicate reasonable lipophilicity for the use of these compounds as drugs [48,49,50] (see Table 7).

Aqueous solubility is an important requirement for any drug intended to be administered orally or parenterally, as a sufficient amount of active ingredients must be administered in a small volume [51]. The predicted LogS values via SwissADME for the selected and pivot molecules are shown in Table 8.

According to Sepay et al. (2020) [51], predicted LogS values between −4 and −6 indicate moderate solubility, between −2 and −4 good solubility and values greater than −6 indicate poor solubility. The predicted LogS value of imatinib was −6.59, indicating that it has poor solubility; consequently, no molecule was excluded from the study based only on this parameter. The tested molecules presented values in the range of good solubility (LMQC01) and poor solubility (LMQC04).

## 3. Material and Methods

### 3.1. Selection of Compounds

In this study, a ligand-based virtual screening was performed on the pivotal compound 4-[(4-methylpiperazin-1-yl)methyl]-N-[4-methyl-3-[(4-pyridin-3-ylpyrimidin-2yl)amino]phenyl]benzamide, imatinib (STI-571), due to its defined crystallography at the active site of the tyrosine kinase enzyme (PDB ID 1IEP). Virtual screening was performed in three commercial compound databases, totaling six libraries (ChemBridge-DIVERSet, ChemBridge-DIVERSet-EXP, Zinc_Drug Database, Zinc_Natural_Stock, Zinc_FDA_BindingDB, Maybridge), using the ROCS V2.4.1 (Rapid Overlay of Chemical Structures—similarity by shape) program [52,53]. The aim was to score the three-dimensional overlaps, comparing the conformation and volume of the compounds in the databases with the reference structures (STI-571) to obtain the “Top2000/base”, totaling 12,000 structures. 

One of the fundamentally important characteristics is the shape of the structures, since it plays a crucial role in molecular selectivity between the ligands and the biological target [54]. Subsequently, ligand-based virtual screening was performed in the EON (electrostatic similarity) software [55], aiming to compare the electrostatic surfaces of the structures selected from the ROCS with the reference structures. The EON program calculated the Tanimoto electrostatic index of the structures from the six databases and the structure of imatinib (STI-571), in addition to calculating new partial charges for the input structures. The results are grouped according to score and classified based on the “ET_combo” analogous to the “ComboTanimoto” [55]. The electrostatic classification based on the “ET_combo” score ranges from 1.0 (identical) to negative values resulting from the overlap of positive and negative charges. Thus, we obtained the “Top100/base”, totaling 600 compounds for performing pharmacokinetic and toxicological predictions, which will be performed with the aid of the QikProp [23] and Derek Nexus [56] programs.

### 3.2. Pharmacokinetic and Toxicological Properties Predictions

The study of the absorption, distribution, metabolism, excretion and toxicity (ADME/Tox) properties for the compounds selected by virtual screening aimed to exclude from the study structures with unsatisfactory pharmacokinetic and toxicological data. The program used in this stage was QikProp [23]. This program made predictions of the pharmacokinetic properties of the 600 structures resulting from the electrostatic similarity screening, analyzing the chemical structure of each molecule as a whole and basing its predictions on the 3D structure.

With the aid of the DEREK NEXUS software, toxicity predictions were made by analyzing the carcinogenicity, mutagenicity, genotoxicity, hepatotoxicity and teratogenicity properties. The program considers data on chemical structures and toxicity reports in the literature to perform a comparison with the groups present in the molecules analyzed [56]. For each group that has something potentially toxic, the program generates an alert (probable, plausible, improbable, possible, certain, impossible, doubtful). At the end of the procedure, an analysis of the structure as a whole and the potential toxicity of the molecule is obtained. Compounds with the best pharmacokinetic and toxicological properties were then selected for a later stage of predicting biological activity.

### 3.3. Biological Activity Prediction

Biological activity prediction was performed using the PASS software [16,17], which is based on the suggestion that the chemical structure of compounds is closely related to their biological activity. The software was used to predict the potential biological activity of the compounds with the best ADME/Tox predictions, and the compounds that had satisfactory results for tyrosine kinase antiproliferative activity and BCR-ABL inhibition were subjected to molecular docking study.

### 3.4. Molecular Docking Study

Molecular docking is a method used to predict the interaction between a small molecule and a protein at the atomic level, which allows us to characterize the behavior of small molecules at the binding site of target proteins, as well as to elucidate fundamental biochemical processes [57]. The docking process involves two basic steps: the prediction of the ligand conformation, as well as its position and orientation within the sites (generally referred to as pose) and the evaluation of the binding affinity [58].

The molecular docking study was carried out using the AutoDock Vina 1.1.2 program [59] and the PyRx 0.8 graphical interface [60]. In this study, the Lamarckian Genetic Algorithm (LGA) was used, with standard parameters of the genetic algorithm (with a population size of 150), a maximum number of evaluations of 250,000, a maximum number of generations of 27,000 and a crossing rate of 0.8, following the protocol of the Applied Computational Chemistry research group at the Federal University of Amapá. In this study, re-docking was performed to validate the methodology.

The crystallographic model selected in the Protein Data Bank (PDB) was tyrosine kinase (BCR-ABL) complexed with imatinib (PDB ID 1IEP), with a resolution of 2.1 Å [38]. To identify the possible ligand-macromolecule combinations, the search algorithm used was the Lamarckian Genetic Algorithm (LGA), which presents the best results in the search for the global minimum [61].

For this purpose, the crystallographic ligand was removed from the complex (PDB ID 1IEP), and subsequently replaced with the original receptor with the coupling parameters validated by calculating the Root Mean Square Deviation (RMSD) to obtain the best conformation, following the previously validated protocol adopted by our research group [62,63,64].

The target protein structure (PDB ID: 1IEP) was downloaded in *PDB format from the RCSB Protein Data Bank [65] and used in the preparation of the ligand and receptor via the Discovery Studio (DS) Visualizer 17.2.0 program [66]. Afterwards, the compounds were coupled and their binding affinity energies were calculated for the target. The DS program was used for the calculation of RMSD and utilized to generate the interactions between the inhibitors and the receptor.

In selecting the best combination of parameters, the location of the conformation with the lowest RMSD in the most populated cluster and with the lowest binding affinity was considered. To visually evaluate the best result obtained from the validation, the conformation obtained by X-ray diffraction (PDB ID 1IEP) was superimposed on the conformation with the lowest RMSD obtained in the docking. After validation, a molecular docking study was performed with the structures selected in the virtual screening.

The spatial coordinates (X, Y and Z) were determined in the active site region according to the observed interaction between the enzymes and their respective original ligands. The coordinates used for the center and the size of the grid can be seen in Table 9. Visualizations as well as distance measurements of the interactions between the ligands and enzymes were performed using the DS program.

### 3.5. Molecular Dynamics Simulations

Molecular dynamics simulations were performed using the Desmond v7.7 module [67,68] of the Schrödinger software package as implemented in Maestro [69]. All complexes were placed in an orthorhombic water box at a buffer distance of 10 Å and solvated with SPC water models. A 0.15 M NaCl salt concentration was added and additional Na^+^/Cl^−^ ions were added to neutralize the systems. The MD was performed in the NPT ensemble at a temperature of 300 K and 1 atm pressure over 300 ns with recording intervals of 1.2 ps for energy and 300 ps for trajectory. 

Simulations were run with the OPLS4 force field. Plots and figures were sketched with the Desmond simulation interaction diagram tool of Maestro. The binding energy between the protein–ligand complexes was calculated over the 300 ns period with thermal_mmgbsa.py python script provided by Schrödinger, which takes a Desmond trajectory file, splits it into individual snapshots, runs the Prime-MMGBSA [70,71,72] calculations on each frame, and yields the average calculated binding energy.

### 3.6. Structure–Activity Relationship (SAR) and Molecular Overlay

The structure–activity relationships (SAR) were realized following methodology similar to that proposed by Ferreira et al. (2019) [73], Silva et al. (2023) [74], and Lima et al. (2022) [47]. A search for the promising compounds was carried out in the PubChem database [41] in order to obtain information related to experimental data, patents or research with such molecules selected in virtual screening [75].

The molecular overlay was realized according to the methodology proposed by Santos et al. (2022) [76] and da Silva Costa et al. (2018) [77]. The chemical structures were superimposed, considering the contributions (%) of steric and electronic fields. The analyses were performed using the DS program with the contributions of 50%, 70% and 100% of the steric field.

### 3.7. Synthetic Accessibility and Theoretical Synthetic Route of Promising Compounds

Synthetic accessibility (SA) is an important factor in the selection of potential bioactive compounds. SwissADME [45,46,47] performs fragment-based SA prediction [76,77,78,79,80,81,82,83] by analyzing more than 13 million compounds. The method used is based on the fact that frequent fragments imply high SA, i.e., easy synthesis, and rare fragments imply low SA, i.e., difficult synthesis. SA scores range from 1 (very easy) to 10 (very difficult).

### 3.8. Lipophilicity and Water Solubility via SwissADME Webserver

This analysis aimed to determine solubility data for promising compounds for future in vitro and in vivo assays, focusing on accurate dilution, solution preparation, and solvent selection. Various methods can estimate LogPo/w for different chemical groups. In this work, we used multiple lipophilicity prediction methods to improve accuracy by allowing selection of the best method or achieving a consensus value through comparison [78].

The SwissADME webserver offers five prediction methods for accurate data on promising compounds for future biological assays. XLOGP3 is an atomic method with corrective factors, using reference LogP values as a baseline [79]. WLOGP is another atomistic method without corrective factors, based on the Wildman and Crippen fragmentation approach [80]. MLOGP uses a topological method that applies multiple linear regression on 13 lipophilicity-related molecular descriptors [81]. SILICOS-IT is a hybrid method combining 27 fragments and 7 topological descriptors via FILTER-IT software [83] (https://www.hydac.com/en/online-tools/filter-it2/, accessed on 12 April 2024). Finally, iLOGP is a physics-based method that calculates the free energy of solvation in n-octanol and water using Born’s implicit solvent equation and solvent-accessible surface area (GB/SA) [47].

Water solubility (LogS) is a key factor in determining compound dilution and appropriate administration. SwissADME offers three topological methods for assessing water solubility: the ESOL method [84], the Ali method [85] and the SILICOS-IT method [82].

## 4. Conclusions

In this comprehensive study of ADME/Tox and the molecular interactions of the studied compounds, together with docking and molecular dynamics simulations, we concluded that the ligands and the protein had low and stable RMSD values, suggesting that the selected compounds form stable complexes and have potential as inhibitors of the tyrosine kinase enzyme encoded by the BCR-ABL gene. Our in silico results showed that the studied molecules could be potent protein kinase inhibitors with potential antiproliferative activity on the enzyme. In conclusion, the results suggest that these ligands, particularly LMQC04, may bind strongly to the studied target and may have appropriate ADME/Tox properties in experimental studies. Considering future in vitro or in vivo assays, we elaborated the theoretical synthetic route of the promising compounds identified in the present study. Based on these findings, the selected ligands showed promise for future studies in developing chronic myeloid leukemia treatments.

## Figures and Tables

**Table 1:** Toxicological alerts for imatinib obtained from Derek Nexus v. 4.1.0 software.

Compound	Toxicity Prediction Alert	Toxic Group	Toxicity Alert
Imatinib	Methemoglobinaemia	Simple Aniline 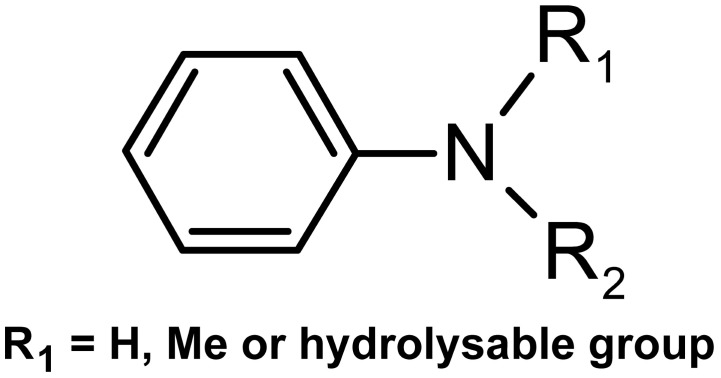	Plausible

**Table 2:** Pharmacokinetic values of compounds obtained by virtual screening that showed biological activity for the purpose of the research.

Compounds resulting from virtual screening based on imatinib	**Entry**	**Compounds**	**#Stars**	**SNC**	**%AOH**	**#Metab**	**Volume**	**QPPCaco**	**QPlogCS**	**HFOAS**	**HFIAS**
	**Reference**	**Imatinib**	3	1	91.058	8	493.610	75.791	−0.391	338.382	95.970
	**LMQC01**	**BindingDB1944**	0	−2	83.755	4	456.562	188.827	−1.755	356.054	181.360
	**LMQC02**	**Omega39040**	0	0	82.665	2	461.451	75.894	−0.561	421.652	125.478
	**LMQC03**	**ZINC29051126**	0	0	100.000	3	489.250	125.654	−0.465	365.621	102.632
	**LMQC04**	**Omega9146**	0	0	100.000	4	459.909	181.654	−0.671	345.025	111.375
	**LMQC05**	**BindingDB50001859**	0	0	87.507	5	385.508	246.411	−0.466	477.384	105.572
	**LMQC06**	**BindingDB31046**	0	0	100.000	5	281.357	1582.196	−0.493	233.195	84.007
	**LMQC07**	**BindingDB50335522**	0	−1	86.157	7	406.536	56.082	−1.202	497.059	158.911
	**LMQC08**	**Omega48308**	0	0	82.759	5	372.423	349.361	−0.291	441.534	89.584
	**LMQC09**	**Omega45294**	0	0	83.631	2	367.468	201.737	−0.262	270.567	114.733

These descriptors must present values in a certain range to verify their potential for later development as a drug. The number of calculated properties that fall outside the required range for 95% of known drugs (#Stars) must be in the recommended range of 0 to 5. Activity in the central nervous system (CNS) follows the scale of −2 (inactive) to +2 (active). The hydrophobic component of the solvent accessible surface area (HFOAS) should remain in the recommended range of 0.0 to 750.0 Å^2^. The hydrophilic component of the solvent accessible surface area (HFIAS) has a recommended range of 7.0 to 330.0 Å^2^. The total volume of the molecule should remain in the range 500 to 2000 Å^3^. Number of hydrogen donors is in the range 0 to 6. The number of hydrogen bonds accepted by the molecule should remain in the range 2–20. The permeability of the Caco-2 cell membrane, in nm/s, should be in the range <5 low, >500 high. The logarithm of the predisposed partition coefficient of the blood-brain barrier should remain in the range −3.0 to 1.0. The number of probable metabolic reactions should be at most 8 and the percentage of human oral absorption above 80% is considered high, and values below 25% indicate low absorption [15].

**Table 3:** Compounds that exhibit biological activity values for tyrosine kinase inhibition activity.

Compound	Structure	Biological Activity	Pa ^[a]^	Pi ^[b]^
**Imatinib**	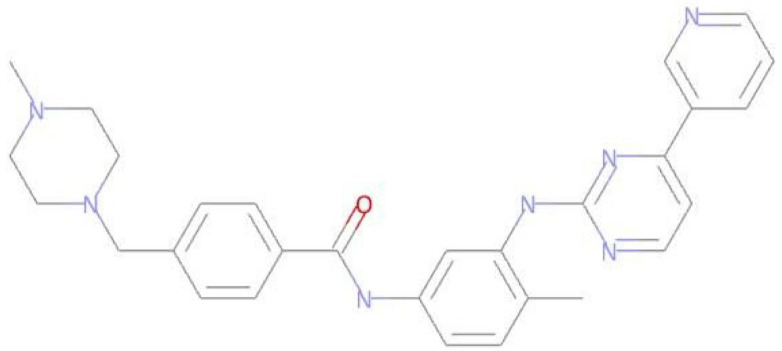	Protein Kinase Inhibitor	0.802	0.005
**LMQC01**	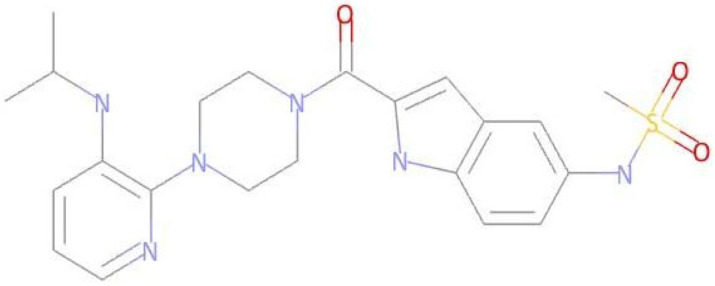		0.457	0.049
**LMQC02**	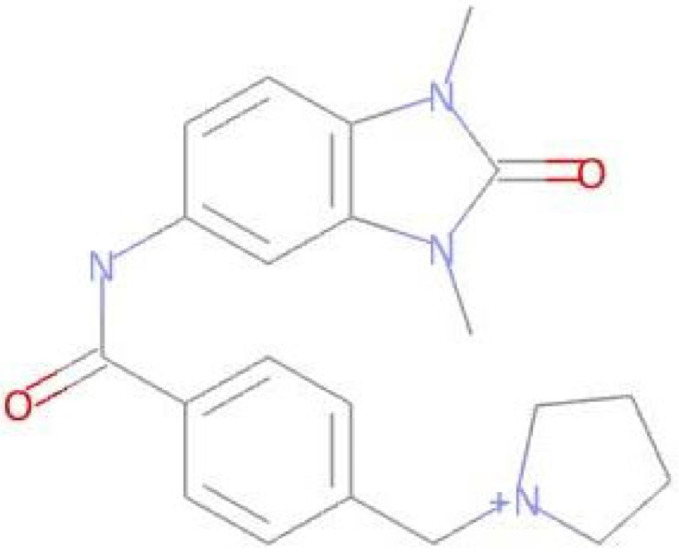		0.137	0.049
**LMQC04**	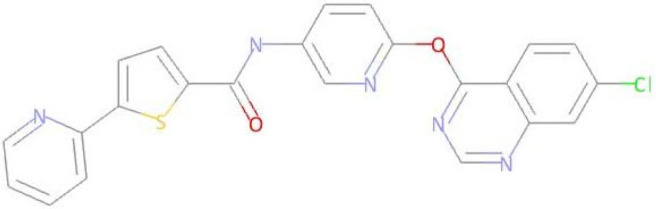		0.658	0.021
**LMQC08**	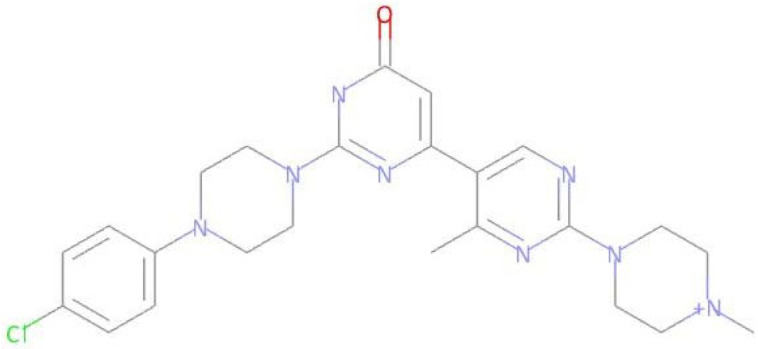		0.222	0.013

^[a]^ Pa = probability of being active (Pa > 0.000 or Pa = 1.000); ^[b]^ Pi = Probability of being inactive (Pi = 0.000).

**Table 4:** Binding affinity values of the studied compounds obtained from the docking simulations against the Ab1 kinase domain.

Ligand	Experimental Binding Affinity (kcal mol^−1^)	Ki (nM)	Predicted Binding Affinity (kcal mol^−1^)
Imatinib	−11.18 ^[a]^	13.0 [38]	−13.3
LMQC01	-	-	−8.6
LMQC04	-	-	−12.2

^[a]^ Values calculated from experimentally determined inhibition constants (Ki), found in the PDBs, according to the equation ΔG = R.T.lnKi [39,40], were R (gas constant) = 1.987 × 10^−3^ kcal/(mol K) and T (temperature) = 310 K.

**Table 5:** Molecular overlap of the LMQC01 and LMQC04 molecules with the pivot molecule (imatinib).

Compound	Pivot Molecule	Molecular Overlay		
		50 est/50 elt	70 est/30 elt	100 est
LMQC01	Imatinib	0.41	0.57	0.82
LMQC04		0.41	0.58	0.83

est: steric contribution; elt: electrostatic contribution.

**Table 6:** Synthetic accessibility values for imatinib, LMQC01 and LMQC04 predicted via SwissADME and AMBIT-SA.

Compound	Synthetic Accessibility Score	
	SwissADME	AMBIT-SA
**Imatinib**	3.78	65.70
**LMQC01**	4.46	58.32
**LMQC04**	3.43	55.26

**Table 7:** Prediction of LogP values for two selected molecules and imatinib via SwissADME and consensus.

Compound	iLOGP	XLOGP3	WLOGP	MLOGP	Silicos-IT LogP	Consensus LogP
Imatinib	4.04	3.52	3.49	2.15	3.69	3.38
LMQC01	2.68	2.11	2.65	0.75	0.53	1.75
LMQC04	3.67	4.77	5.66	2.06	5.29	4.29

**Table 8:** Prediction of LogS values for two selected molecules and imatinib via Swiss ADME and consensus.

Compound	ESOL LogS	Ali LogS	Silicos-IT LogSw	Consensus LogS
Imatinib	−5.07	−5.02	−9.67	−6.59
LMQC01	−3.88	−4.24	−5.75	−4.62
LMQC04	−5.92	−6.98	−9.63	−7.51

**Table 9:** Protocol used in the validation of the molecular docking study.

Enzyme	Inhibitor	Spatial Coordinates of the Grid Center	Grid Dimensions (Angstrom)
BCR-ABL Tyrosine Kinase (PDB ID 1IEP)	Imatinib	X = 14.79	X = 16.5
		Y = 52.87	Y = 25.0
		Z = 15.94	Z = 20.47

## Data Availability

Data is contained within the article or Supplementary Materials.

## Supplementary Materials

The following supporting information can be downloaded at: https://www.mdpi.com/article/10.3390/ph17111491/s1.
