# Supplementary material for: Rational Approach to New Chemical Entities with Antiproliferative Activity on Ab1 Tyrosine Kinase Encoded by the BCR-ABL Gene: An Hierarchical Biochemoinformatics Analysis
Source: Pharmaceuticals (Basel). 2024 Nov 6;17(11):1491. doi: 10.3390/ph17111491 (PMC11597596; doi:10.3390/ph17111491)
Supplement: Supplementary file 1 [file pharmaceuticals-17-01491-s001.zip › pharmaceuticals-3210041-supplementary.pdf]

**SUPPLEMENTARY MATERIALS**

**Figure S1**. Predicted interactions between the compound LMQC01 vs BCR-ABL tyrosine kinase active site

**Figure S2**. Predicted interactions between the compound LMQC04 vs BCR-ABL tyrosine kinase active site

**Figure S3**. Summary of the secondary structure elements (SSE) composition, Alpha-helices (magenta), and beta-strands (blue), for each trajectory frame over the course of the simulation, and SSE distribution by residue assignment in imatinib **(a)**, LMQC01 **(b)**, and LMQC04 **(c)** complexes in MD simulations over 300 ns.

**Figure S4**. Ligand Root Mean Square Fluctuation (L-RMSF) for imatinib **(a)**, LMQC01 **(b)**, and LMQC04 **(c)** complexes. This is useful for characterizing changes in the ligand atom positions.

**Figure S5**. Timeline representation of the interactions and contacts (H-bonds, Hydrophobic, Ionic, Water bridges) for Imatinib **(a)**, LMQC01 **(b)**, and LMQC04 **(c)** complexes. The top panel shows the total number of specific contacts the protein makes with the ligand over the course of the trajectory. The bottom panel shows which residues interact with the ligand in each trajectory frame. Some residues make more than one specific contact with the ligand, which is represented by a darker shade of orange, according to the scale to the right of the plot.

**Figure S6**. Ligand torsions plot for Imatinib **(a)**, LMQC01 **(b)**, and LMQC04 **(c)** complexes. Summarizes the conformational evolution of every rotatable bond (RB) in the ligand throughout the simulation trajectory (0.00 through 300.30 nsec). The top panel shows the 2d schematic of a ligand with color-coded rotatable bonds. Each rotatable bond torsion is accompanied by a dial plot and bar plots of the same color. Dial (or radial) plots describe the conformation of the torsion throughout the course of the simulation. The bar plots summarize the data on the dial plots, by showing the probability density of the torsion. The values of the potential are on the left Y-axis of the chart, and are expressed in kcal/mol. Looking at the histogram and torsion potential relationships may give insights into the conformational strain the ligand undergoes to maintain a protein-bound conformation.

**Figure S7**. Ligand Properties for Imatinib **(a)**, LMQC01 **(b)**, and LMQC04 **(c)** complexes. Ligand RMSD: Root mean square deviation of a ligand with respect to the reference conformation (typically the first frame is used as the reference and it is regarded as time t=0). Radius of Gyration (rGyr): Measures the 'extendedness' of a ligand, and is equivalent to its principal moment of inertia. Intramolecular Hydrogen Bonds (intraHB): Number of internal hydrogen bonds (HB) within a ligand molecule. Molecular Surface Area (MolSA): Molecular surface calculation with 1.4 Å probe radius. This value is equivalent to a van der Waals surface area. Solvent Accessible Surface Area (SASA): Surface area of a molecule accessible by a water molecule. Polar Surface Area (PSA): Solvent accessible surface area in a molecule contributed only by oxygen and nitrogen atoms.

**Table S1.** Interactions with amino acid residues and respective distances of Imatinib

**Table S2.** Interactions with amino acid residues and respective distances of the compound LMQC01

**Table S3.** Interactions with amino acid residues and respective distances of the compound LMQC04

**Table S4**. Prime MM-GBSA energies (kcal $mol^{-1}$) for ligands binding at the Abl-kinase domain. [a]Total free energy of binding, in kcal/mol as calculated by the MMGBSA method, averaged over the time of simulation. [b]Electrostatic Coulomb term of the binding energy. [c]Covalent term of the binding energy. [d]Hydrogen bond contribution to the binding energy. [e]Lipophilic contribution to the binding energy. [f]π–π packing correction. [g]Generalized Born term of the solvation energy. [h]van der Waals term of the binding energy.

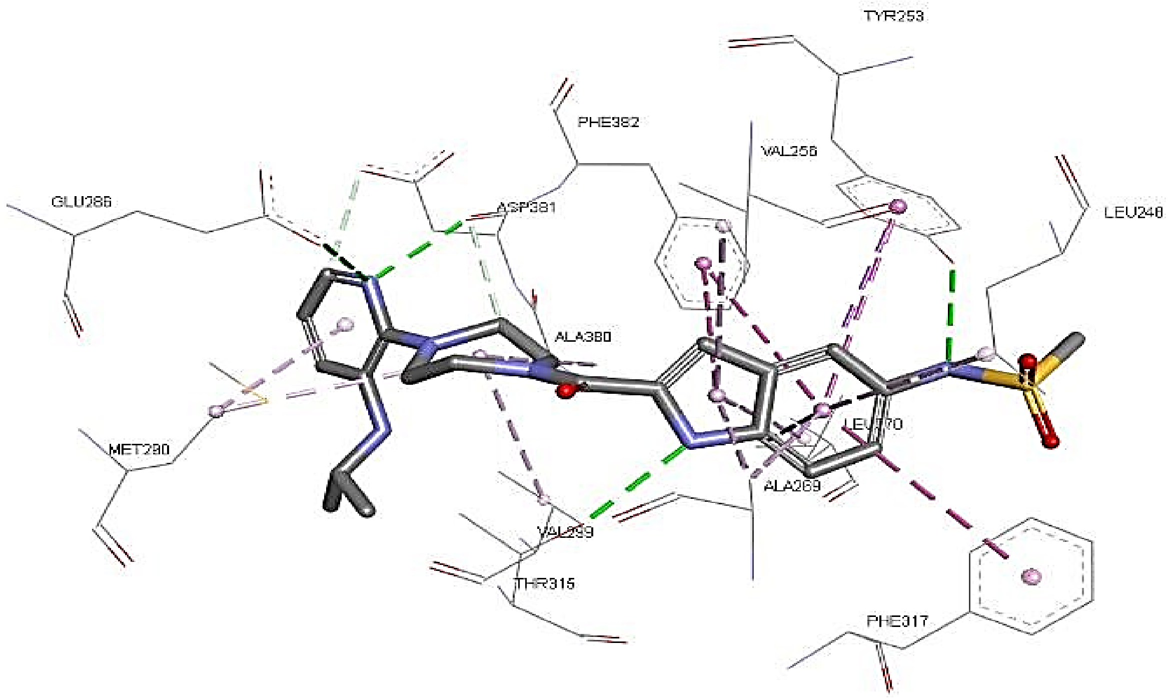


**Figure S1**. Predicted interactions between the compound LMQC01 vs BCR-ABL tyrosine kinase active site.

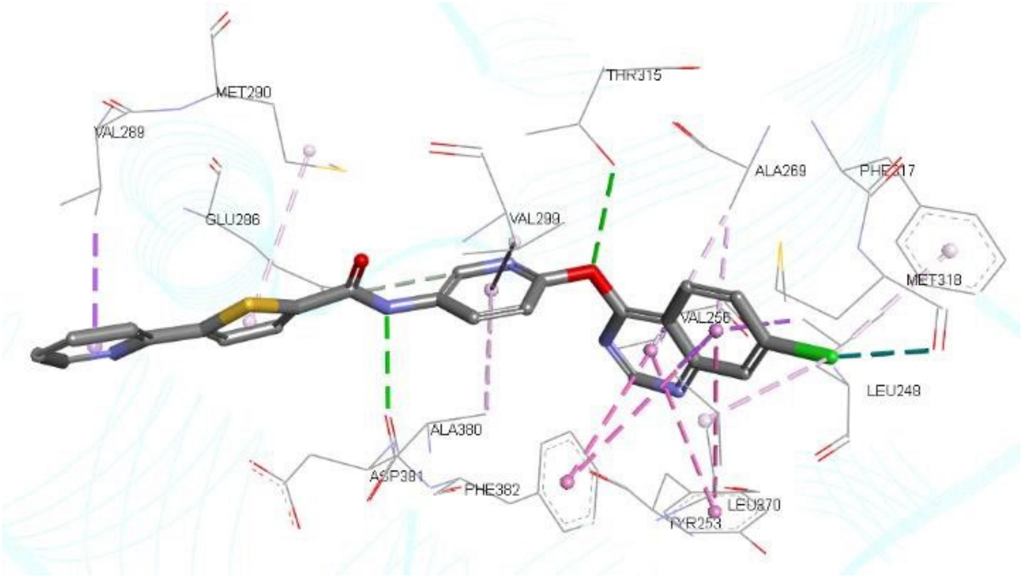


**Figure S2**. Predicted interactions between the compound LMQC04 vs BCR-ABL tyrosine kinase active site.

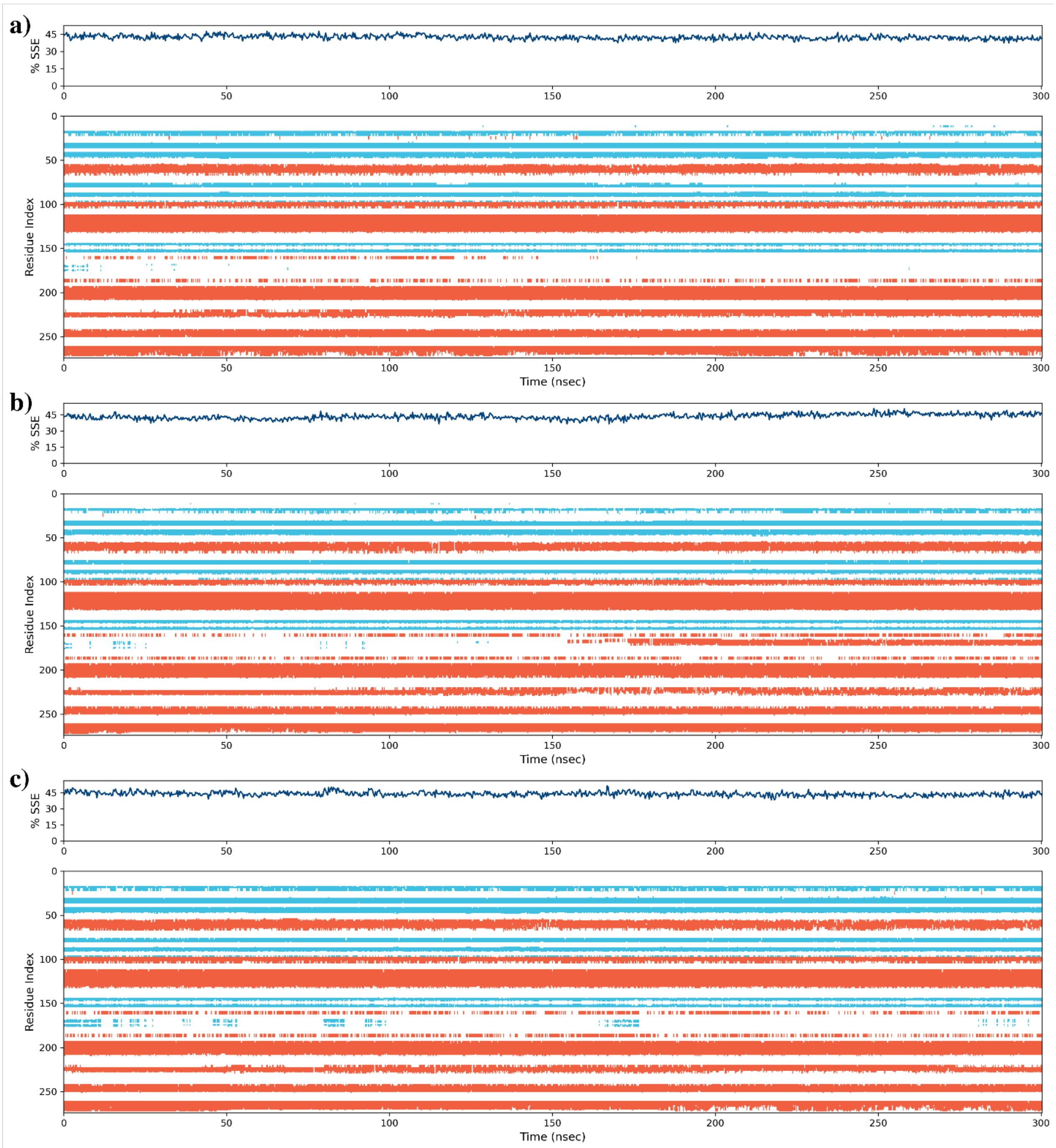


**Figure S3**. Summary of the secondary structure elements (SSE) composition, Alpha-helices (magenta), and beta-strands (blue), for each trajectory frame over the course of the simulation, and SSE distribution by residue assignment in imatinib **(a)**, LMQC01 **(b)**, and LMQC04 **(c)** complexes in MD simulations over 300 ns.

a)

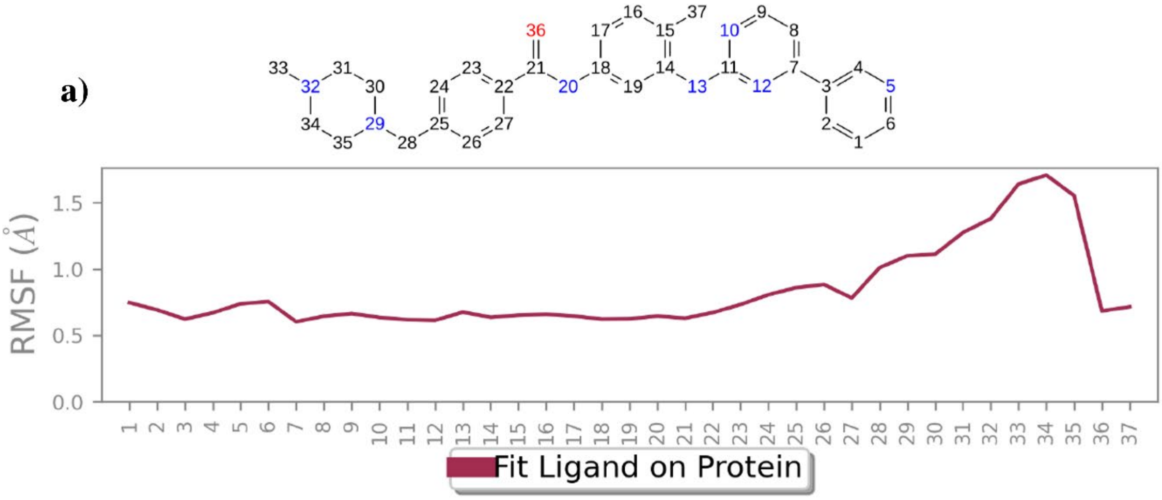


b)

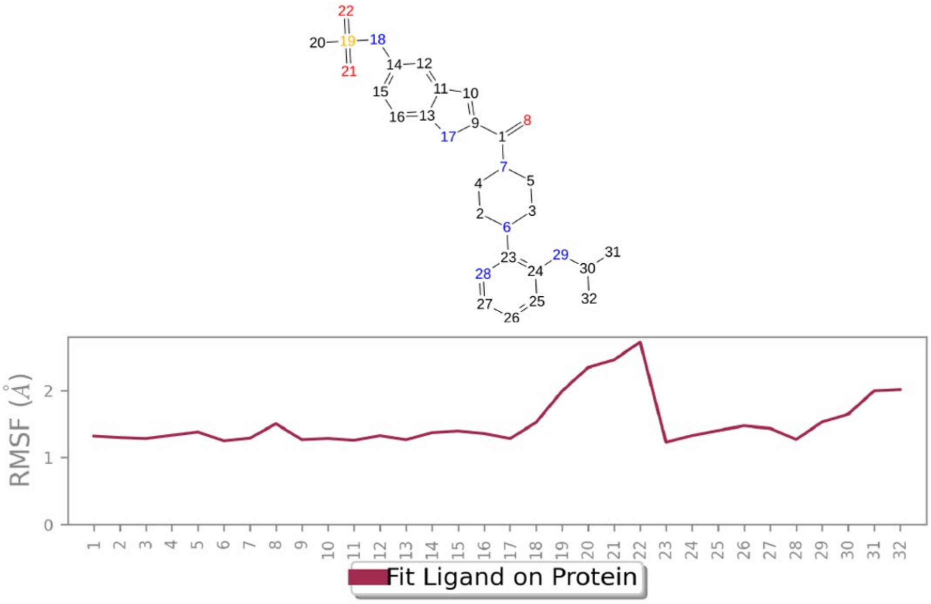


c)

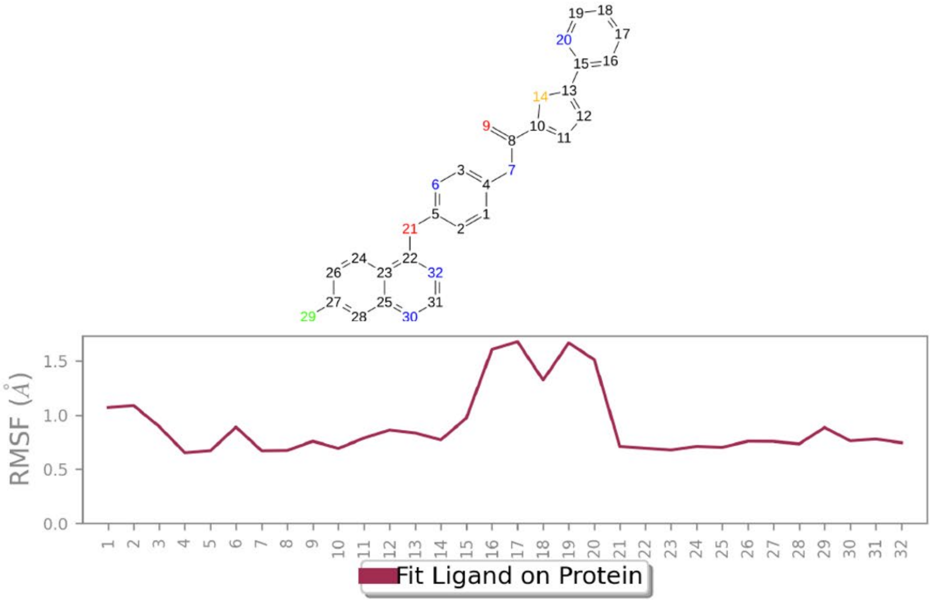


**Figure S4**. Ligand Root Mean Square Fluctuation (L-RMSF) for imatinib **(a)**, LMQC01 **(b)**, and LMQC04 **(c)** complexes. This is useful for characterizing changes in the ligand atom positions.

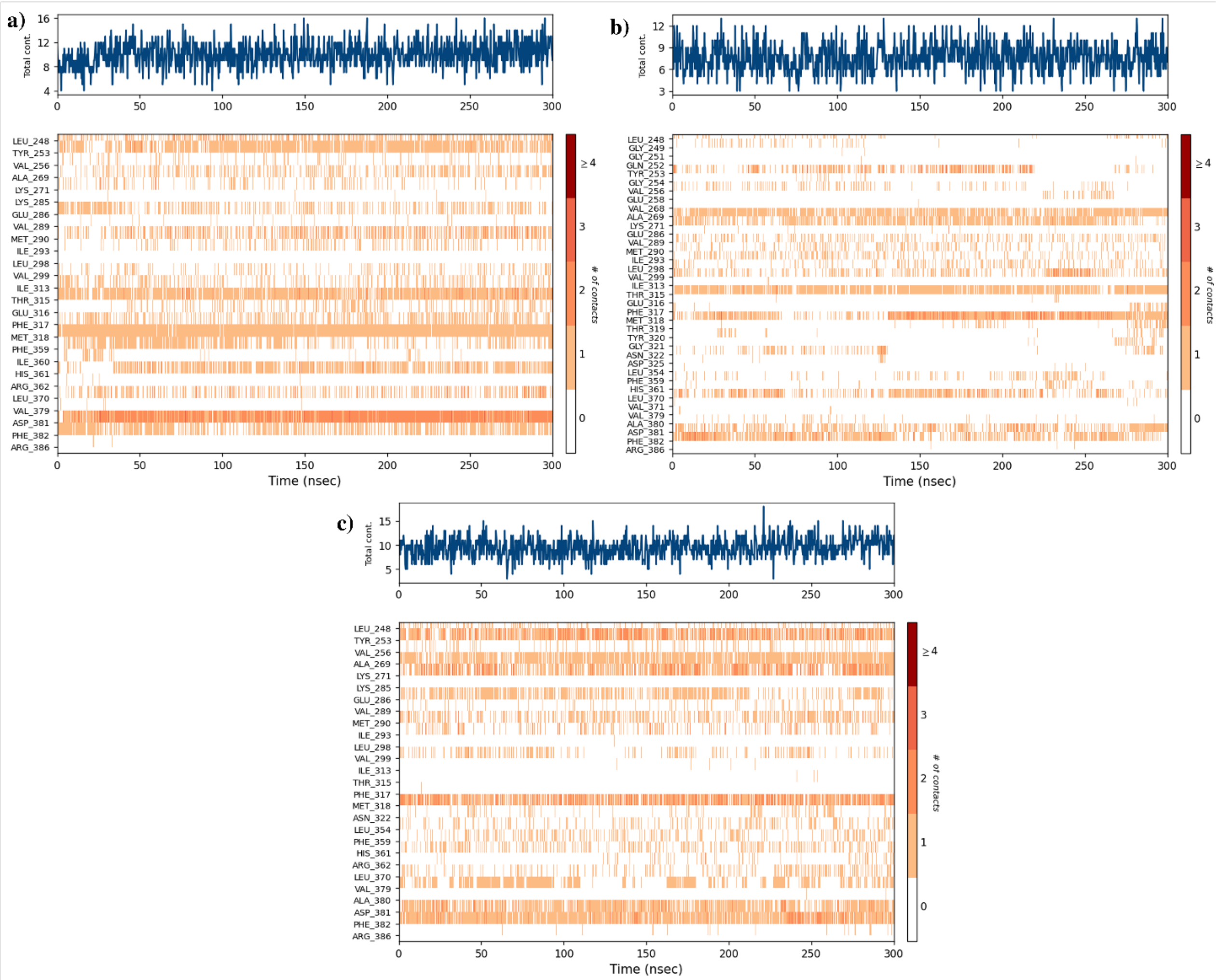


**Figure S5**. Timeline representation of the interactions and contacts (H-bonds, Hydrophobic, Ionic, Water bridges) for Imatinib **(a)**, LMQC01 **(b)**, and LMQC04 **(c)** complexes. The top panel shows the total number of specific contacts the protein makes with the ligand over the course of the trajectory. The bottom panel shows which residues interact with the ligand in each trajectory frame. Some residues make more than one specific contact with the ligand, which is represented by a darker shade of orange, according to the scale to the right of the plot.

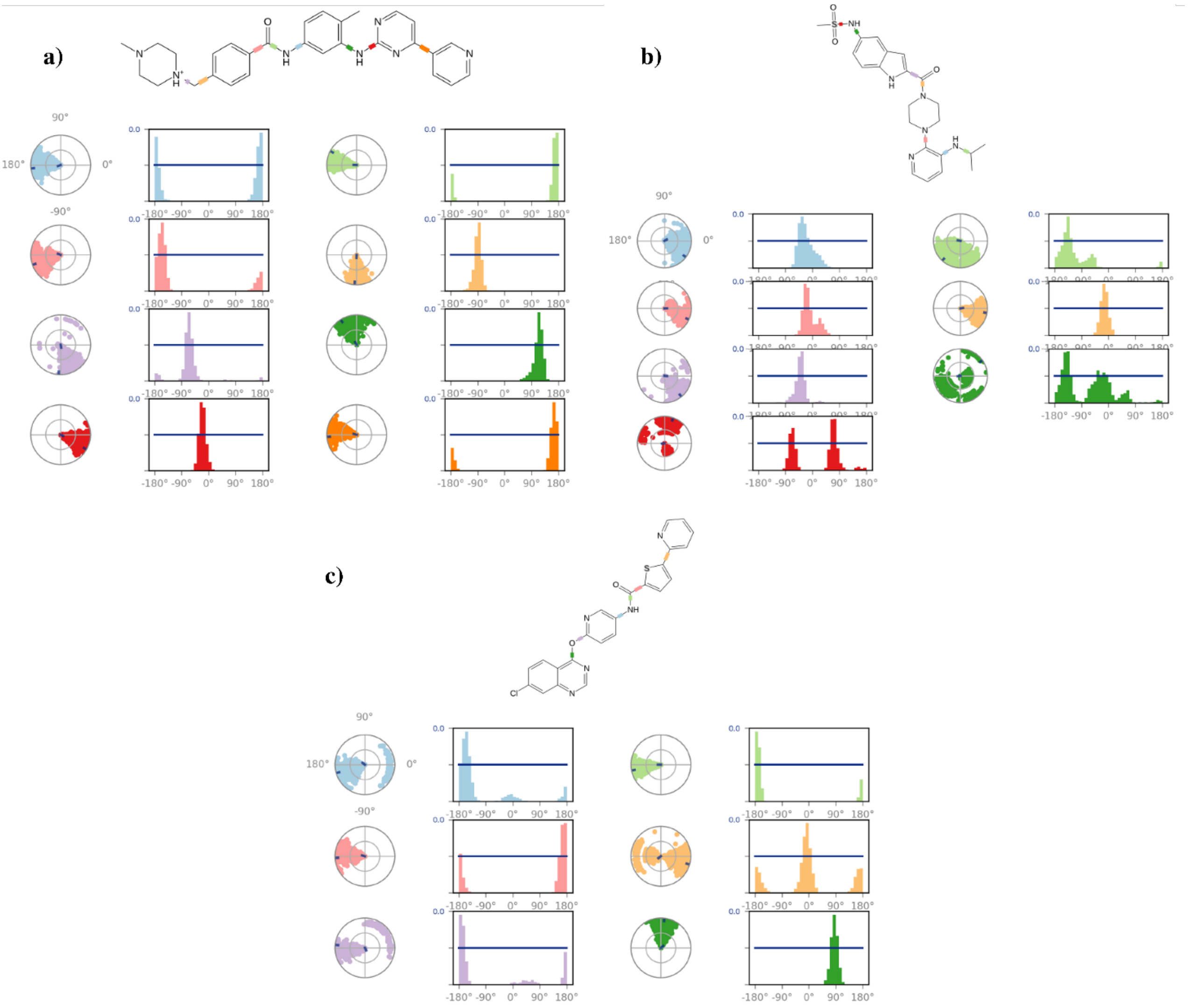


**Figure S6**. Ligand torsions plot for Imatinib **(a)**, LMQC01 **(b)**, and LMQC04 **(c)** complexes. Summarizes the conformational evolution of every rotatable bond (RB) in the ligand throughout the simulation trajectory (0.00 through 300.30 nsec). The top panel shows the 2d schematic of a ligand with color-coded rotatable bonds. Each rotatable bond torsion is accompanied by a dial plot and bar plots of the same color. Dial (or radial) plots describe the conformation of the torsion throughout the course of the simulation. The bar plots summarize the data on the dial plots, by showing the probability density of the torsion. The values of the potential are on the left Y-axis of the chart, and are expressed in kcal/mol. Looking at the histogram and torsion potential relationships may give insights into the conformational strain the ligand undergoes to maintain a protein-bound conformation.

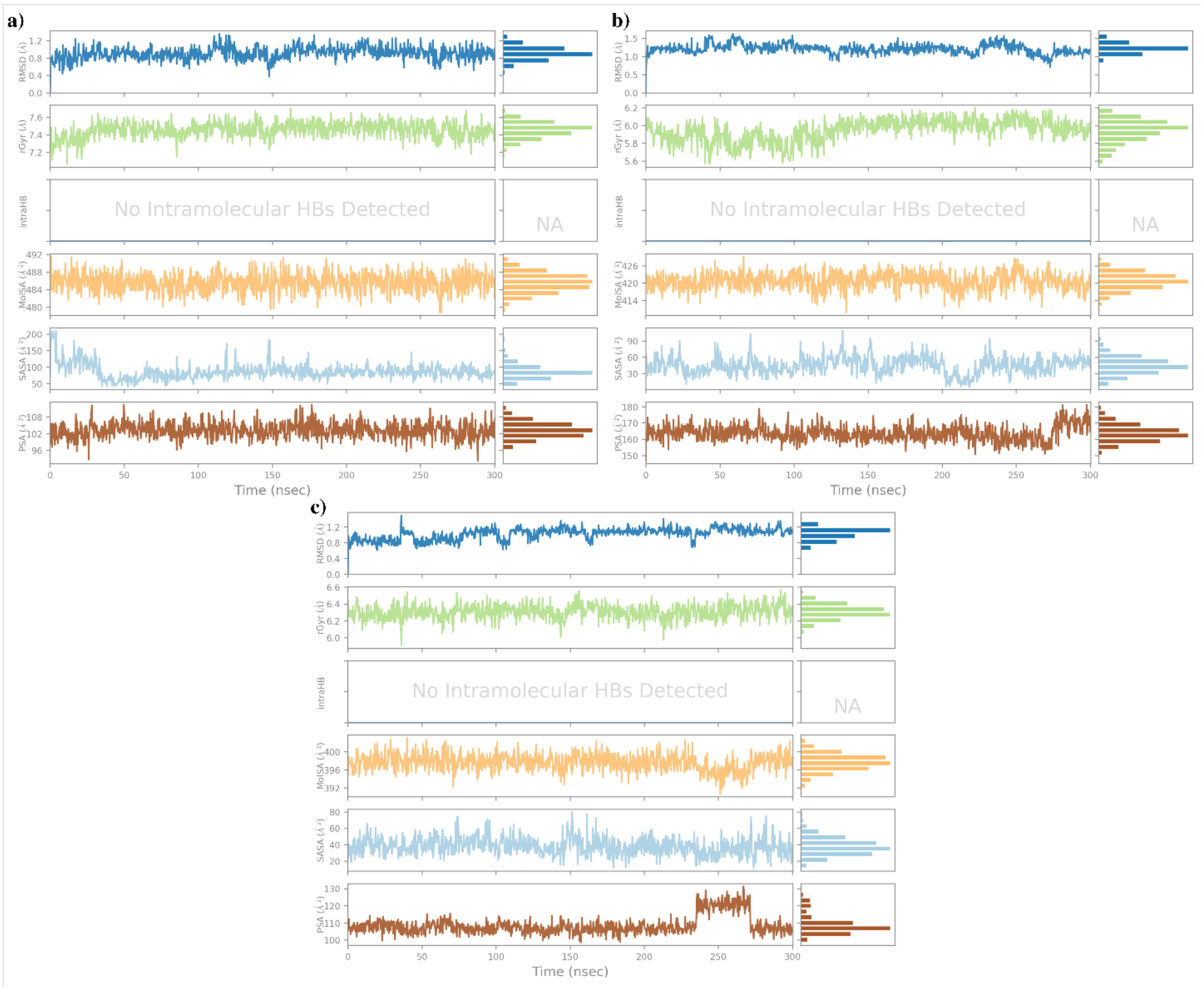


**Figure S7**. Ligand Properties for Imatinib **(a)**, LMQC01 **(b)**, and LMQC04 **(c)** complexes. Ligand RMSD: Root mean square deviation of a ligand with respect to the reference conformation (typically the first frame is used as the reference and it is regarded as time t=0). Radius of Gyration (rGyr): Measures the 'extendedness' of a ligand, and is equivalent to its principal moment of inertia. Intramolecular Hydrogen Bonds (intraHB): Number of internal hydrogen bonds (HB) within a ligand molecule. Molecular Surface Area (MolSA): Molecular surface calculation with 1.4 Å probe radius. This value is equivalent to a van der Waals surface area. Solvent Accessible Surface Area (SASA): Surface area of a molecule accessible by a water molecule. Polar Surface Area (PSA): Solvent accessible surface area in a molecule contributed only by oxygen and nitrogen atoms.

**Table S1**. Interactions with amino acid residues and respective distances of Imatinib.

| Compounds | Amino acids | Distance (Å) | Type | Binding free energy (kcal/mol) |
|---|---|---|---|---|
| Imatinib vs Abl kinase domain | Leu-248 | 3.90 | π -Sigma | -13.3 |
| | Tyr-253 | 5.53 | π - π T-shaped | |
| | Tyr-253 | 5.00 | π - π T-shaped | |
| | Val-256 | 4.72 | Alkyl | |
| | Val-256 | 4.58 | π -alkyl | |
| | Ala-269 | 4.39 | π -alkyl | |
| | Ala-269 | 5.12 | π -alkyl | |
| | Ala-269 | 4.39 | π -alkyl | |
| | Ala-269 | 4.06 | Alkyl | |
| | Lys-271 | 4.89 | π -alkyl | |
| | Glu-286 | 2.99 | hydrogen bonds | |
| | Val-289 | 5.46 | Alkyl | |
| | Met-290 | 5.04 | π -alkyl | |
| | Met-290 | 3.30 | hydrogen bonds | |
| | Tyr-315 | 2.88 | hydrogen bonds | |
| | Phe-317 | 4.63 | π - π stacked | |
| | Met-318 | 2.90 | hydrogen bonds | |
| | Ile-360 | 2.93 | hydrogen bonds | |
| | Leu-370 | 4.42 | π -alkyl | |
| | Asp-381 | 3.45 | hydrogen bonds | |
| | Asp-381 | 5.12 | π -alkyl | |

**Table S2**. Interactions with amino acid residues and respective distances of the compound LMQC01.

| Compounds | Amino acids | Distance (Å) | Type | Binding free energy (kcal/mol) |
|---|---|---|---|---|
| LMQC01 vs Abl kinase domain | Leu-248 | 5.37 | π -alkyl | -8.6 |
| | Tyr-253 | 3.10 | hydrogen bonds | |
| | Tyr-253 | 5.27 | π -alkyl | |
| | Tyr-253 | 3.80 | π -sigma | |
| | Val-256 | 5.41 | π -alkyl | |
| | Ala-269 | 4.40 | alkyl | |
| | Ala-269 | 3.64 | π -alkyl | |
| | Glu-286 | 2.83 | hydrogen bonds | |
| | Met-290 | 5.46 | π -alkyl | |
| | Met-290 | 4.37 | π -alkyl | |
| | Val2-99 | 4.77 | π -alkyl | |
| | Thr-315 | 2.91 | hydrogen bonds | |
| | Phe-317 | 5.86 | π - π stacked | |
| | Ala-380 | 5.13 | alkyl | |
| | Asp-381 | 2.73 | hydrogen bonds | |
| | Asp-381 | 3.12 | H-C bond | |
| | Asp-381 | 3.54 | H-C bond | |
| | Phe-382 | 5.40 | π -alkyl | |
| | Phe-382 | 5.07 | π -alkyl | |

**Table S3**. Interactions with amino acid residues and respective distances of the compound LMQC04.

| Compounds | Amino acids | Distance (Å) | Type | Binding free energy (kcal/mol) |
|---|---|---|---|---|
| LMQC04 vs Abl kinase domain | Leu-248 | 3.99 | π - sigma | -12.2 |
| | Tyr-253 | 4.84 | π - π stacked | |
| | Tyr-253 | 5.60 | π - π stacked | |
| | Val-256 | 3.82 | π - sigma | |
| | Ala-269 | 4.34 | π -alkyl | |
| | Ala-269 | 3.83 | π -alkyl | |
| | Glu-286 | 3.22 | hydrogen bonds | |
| | Glu-286 | 3.53 | H-C bond | |
| | Val-289 | 3.74 | π - sigma | |
| | Met-290 | 4.93 | π -alkyl | |
| | Val-299 | 4.63 | alkyl | |
| | Tyr-315 | 3.10 | hydrogen bonds | |
| | Phe-317 | 4.12 | π -alkyl | |
| | Met-318 | 3.20 | Halogen | |
| | Leu-370 | 4.31 | π - sigma | |
| | Ala-380 | 4.90 | π -alkyl | |
| | Asp-381 | 3.11 | hydrogen bonds | |
| | Phe-382 | 4.46 | π - π stacked | |
| | Phe-382 | 5.46 | π - π stacked | |

**Table S4**. Prime MM-GBSA energies (kcal $mol^{-1}$) for ligands binding at the Abl-kinase domain. [a]Total free energy of binding, in kcal/mol as calculated by the MMGBSA method, averaged over the time of simulation. [b]Electrostatic Coulomb term of the binding energy. [c]Covalent term of the binding energy. [d]Hydrogen bond contribution to the binding energy. [e]Lipophilic contribution to the binding energy. [f]π–π packing correction. [g]Generalized Born term of the solvation energy. [h]van der Waals term of the binding energy.

| Ligand | [a]$\Delta G_{bind}$ | [b]$\Delta G_{Coulomb}$ | [c]$\Delta G_{Covalent}$ | [d]$\Delta G_{Hbond}$ | [e]$\Delta G_{Lipo}$ | [f]$\Delta G_{Packing}$ | [g]$\Delta G_{SolvGB}$ | [i]$\Delta G_{vdW}$ |
|---|---|---|---|---|---|---|---|---|
| **Imatinib** | -101.03 ±5.28 | -82.89 | 2.68 | -1.55 | -35.81 | -2.47 | 95.69 | -75.30 |
| **LMQC01** | -69.00 ±6.01 | -16.76 | 2.74 | -1.04 | -20.71 | -1.14 | 66.96 | -66.04 |
| **LMQC04** | -83.25 ±4.09 | -14.56 | 1.82 | -0.65 | -29.46 | -3.40 | 27.86 | -64.86 |
